# The effect of vitamin D on insulin resistance in patients with type 2 diabetes

**DOI:** 10.1186/1758-5996-5-8

**Published:** 2013-02-26

**Authors:** Afsaneh Talaei, Mahnaz Mohamadi, Zahra Adgi

**Affiliations:** 1 Thyroid Disorders Research Center, Arak University of Medical Sciences, Arak, Iran; 2 Department of Endocrinology, Arak University of Medical Sciences, Arak, Iran

**Keywords:** Diabetes, Insulin resistance, Vitamin D

## Abstract

**Introduction:**

Over the past decade, numerous non-skeletal diseases have been reported to be associated with vitamin D deficiency including type2 diabetes mellitus (T2DM). Different studies provide evidence that vitamin D may play a functional role in glucose tolerance through its effects on insulin secretion and insulin sensitivity. This study evaluates the effects of vitamin D supplementation on insulin resistance in T2DM.

**Method:**

Through a before-after study, 100 patients with T2DM, 30–70 years old, were recruited from an Arak diabetes clinic as consecutive attenders. Participants were assessed for clinical and biochemistry. Serum insulin and, 25(OH)D concentration, and HOMA-IR was calculated. All measurements were performed at the beginning and the end of the study. Patients received 50,000 unit of vitamin D_**3**_ orally per week for eight weeks, Statistical analysis was made using SPSS17. The results were analyzed by descriptive tests, and a comparison between variables were made using paired T-tests or Wilcoxon tests, as appropriate.

**Results:**

100 participants including 70 women (70%) and 30 men (30%) took part in the study. All results were presented as Mean±SD, or medians of non-normally distributed.

24% of the participants were Vitamin D deficient {serum 25(OH)D ≤ 20 ng/ml(50 nmol/l)}.

Mean serum 25 (OH) D concentration was 43.03± 19.28 ng/ml (107.5±48.2 nmol/l).

The results at baseline and at the end, for FPG were 138.48±36.74 and 131.02±39 mg/dl (P=0.05), for insulin, 10.76±9.46 and 8.6±8.25 μIu/ml (P=0.028) and for HOMA-IR, 3.57±3.18 and 2.89±3.28 (P=0.008) respectively.

**Conclusion:**

Our data showed significant improvements in serum FPG, insulin and in HOMA-IR after treatment with vitamin D, suggested that vitamin D supplementation could reduce insulin resistance in T2DM.

## Introduction

Over recent decades, numerous non-skeletal diseases associated with vitamin D deficiency have been reported including T2DM(type2 diabetes melitus) [1].

T2DM and vitamin D deficiency have risk factors in common such as American-African race, obesity, aging and low physical activity [2]. Also there are associations of vitamin D deficiency with diseases such as osteoporosis, cardiovascular disease and metabolic syndrome disorders diseases [3-5].

Some studies have shown a relationship between vitamin D deficiency and T2DM [6]. Also some other studies have shown that vitamin D may play a functional role on glucose tolerance through its effects on insulin secretion and insulin sensitivity [7].

In comparison to healthy controls, subjects with T2DM have significantly lower circulating concentration of 25 (OH)D [8]. Also the prevalence of vitamin D deficiency in women with T2DM is more common and also,old men with vitamin D deficiency, secret higher insulin after glucose intake [9,10].

Animal studies have shown that vitamin D is a basic factor, necessary for normal insulin secretion [11,12].Vitamin D reduces insulin resistance probably through its effect on calcium and phosphorus metabolism and through up regulation of the insulin receptor gene [13].

One study on 5,677 subjects with impaired glucose tolerance showed that vitamin D supplementation increased insulin sensitivity by 54% [14]. Other studies also found that increased vitamin D intake improves insulin sensitivity [15,16].

Another study on 126 healthy people showed that there is a direct relation between insulin sensitivity and 25(OH)D level and that vitamin D deficiency had a negative effect on β-cell function in pancreatic β-cells [17]. One follow-up study, through 20 years on 4,843 patients with T2DM, showed that vitamin D intake was associated with reduced prevalence of the T2DM [18].

T2DM is considered to develop from a state of increased insulin resistance and β-cell dysfunction develope [19]. Studies on associations between insulin secretion and serum 25 (OH)D have been inconsistent. We have evaluated the effects of vitamin D supplementation on insulin resistance in subjects with T2DM.

## Methods

Through a before and after matched, single blind study, 100 patients with T2DM aged 30–70 years old, were on a diet (more intake of vegetables and fruits and limited use of bread and rice that are usual foods in Iran) or taking oral hypoglycemic agents(glibenclamide or repaglinide+metformin or only metformin), reffered a diabetes clinic at Arak medical university hospital, participated in the study following invitation of consecutive clinical attenders. The patients took part in the study for eight weeks. We added vitamin D_**3**_ to their medication and recorded their demographic data and medication before and after supplementation. During the trial, the subjects were instructed not to change their diabetes drugs or diet.Participants were assessed for weight, height, and BMI. FPG(Fasting Plasma Glucose) and HbA_**1**_ were measured by enzymatic and chromatographic methods using commercial kits (Bio-system S.A and Human Germany) respectively. We also measured serum creatinine, lipid profiles {TC(Total Cholesterol), TAG(Triacylglyceride), HDL(High Density Lipoprotein) and LDL(Low Density Lipoprotein)}, insulin, Ca(Calcium), P(Phosphorous) and ALP(Alkaline Phosphatase). Serum 25(OH)D was measured by radioimmunoassay(RIA) (kits manufactured by Biosource Europe SA, Belgium). HOMA-IR (Hemostatic model assessment-Insulin resistance) was calculated based on following formula [20]:

$$\text{HOMA}-\text{IR}:\frac{\text{FPG}\left(\text{mmol}/L\right)\times \text{Insulin}\left(\text{μ}\text{Iu}/\text{ml}\right)}{22.5}$$

We also assessed liver function by measuring serum concentration of AST (Aspartate Aminotransferase) and ALT (Alanine Aminotransferase) to rule out liver disease and major non alcoholic fatty disease of the liver as exclusion criteria that might affect vitamin D metabolism.

Inclusion criteria were absence of hepatic, renal and bone diseases, malignancy, any history of the use of drugs such as insulin, anticonvulsants, calcium, vitamin D and an HbA_1_c <8% for the last three months.

Written consent was obtained from all participants. After baseline assessment all patients took 50,000 units of vitamin D_**3**_ weekly, for 2 month. During the treatment, all patients were visited and interviewed about possible side effects, and to determine the degree of compliance. After 2 months of treatment, all laboratory tests and clinical evaluations were repeated as per the initial visit. Patient compliance was assessed by tablet counts at each visit in reports.

The Medical Ethics Committee of Arak medical university approved the study protocol which complied with the current version of the Declaration of Helsinki. Statistical analysis was performed using Statistics Package for Social Science (SPSS version 13, SPSS Inc., Chicago, IL, USA). The Data were analyzed by descriptive tests such as mean, SD(standard deviation), and SE (Standard Error) and K-S (Kolmogorov-Smirnov) tests were performed to assess the normality of the variables before further statistical analysis. All data in this study are presented as mean± SD. The effects of Vitamin D supplementation on the variables were analyzed by paired *t* test(for normally distributed) or Wilcoxon test(for non-normally distributed). Step-wise linear regression analysis was used to provide models for predicting of FPG, insulin and HOMA-IR, after vitamin D supplementation. Baseline 25(OH)D was used in the models.

## Results

100 patients (70 women and 30 men) participated in this study. The mean age of the participants was 54.1±11 years old. The mean weight of the patients at baseline was 70±12 and at the end was 71±05 kg that doesn,t have a meaningful differences. 92% were controlled with oral hypoglycemic agents either as monotheraphy or in combination theraphy (including glibenclamide, metformin and repaglinide) and 8% were on alone diet. Mean 25 (OH) D concentration was 43.03±19.28 ng/ml(107.5±48.2 nmol/l) at baseline and 24% of subjects at baseline were vitamin D deficient based on 25 (OH) D<20 ng/ml(50 nmol/l). FPG before and after treatment was normally distributed,so it was analyzed by paired *t* test. But HOMA-IR and insulin before and treatment were non-normally distributed, so they were analyzed by Wilcoxon test. FPG and insulin concentration decreased significantly after treatment with vitamin D_**3**_(P=0.05). Comparison of mean for HOMA-IR before and after treatment with vitamin D showed a meaningful reduction after supplementation (Table 1). We stratified baseline serum 25(OH)D and reported the changes in each variable for each stratum by plotting the changes in each variable (Table 2).

**Table 1:** Comparison of biochemical characteristics(mean ± SD) in patients with diabetes type 2 before and after treatment with vitamin D for 8 weeks

**Variable**	**Before treatment**	**After treatment**	**P-value**
**FPG**(mg/dl)	138.48±36.74	131.02±39	0.05
**(mmol/l)**	7.6±2.04	7.27±2.16	
**Insulin**(μIu/ml)	10.76±8.9	8.6±8.25	0.02
**HOMA-IR**	3.57±3.18	2.89±3.28	0.008
**25(OH)D** (ng/ml)	43.03±19.28	60.12±17.2	0.02
**(nmol/l)**	107.5±48.2	150.3±43	

**Table 2:** The effects of vitamin D supplementation on FBS, insulin and HOMA- IR at different baseline vitamin D concentration (Drop line plot of vitamin D)

**Variable**	**Vitamin D <20 (n=20)**			**Vitamin D 20–30 (n=10)**			**Vitamin D 30–45 (n=15)**		
	**Before**	**after**	**sig**	**Before**	**after**	**sig**	**Before**	**after**	**sig**
FBS (mg/dl)	154±31.9	148±44.9	0.4	146±42.3	144±33.6	0.9	133±32.8	138±36.3	0.4
Insulin (μIu/ml)	10.16±9.2	9.1±6.1	0.6	8.7±10.1	14.5±15.5	0.3	11.5±10.3	9.3±8.6	0.4
HOMA_IR	3.6±1.2	3.05±1.6	0.6	3.2±4.05	5.8±6.4	0.2	3.8±3.7	3.5±3.6	0.6
**Variable**	Vitamin D 40–60 (n=45)				Vitamin D>60(n=10)				
	before	after	sig		before	after		sig	
FBS(mg/dl)	131±34.6	120±35.6	0.02		146±48.3	126±43.6		0.3	
Insulin(μIu/ml)	11.8±9.8	7.5±7	0.006		7.4±6.09	5.7±3.3		0.9	
HOMA_IR	3.6±3.05	2.2v2.6	0.001		2.6±2.09	1.79±1.38		0.4	

Eight weeks of vitamin D supplementation did n,t change mean serum concentration of TC,TG, HDL or LDL cholesterol, vs baseline (Tables 3).

**Table 3:** Comparison of lipid profile in patients with diabetes type 2 before and after treatment with vitamin D(mean ± SD) for 8 weeks

**Variable** (mg/dl)	**Before treatment**	**After treatment**	**P-value**
**T-COL**	191.1±32.2	180.2±31.0	0.3
**TAG**	234.4±73.3	201.0±65.1	0.2
**LDL-C**	109.5±26.4	103.0±23.2	0.5
**HDL-C**	42.5±8.1	38.2±7.2	0.3

By using linear regression analysis,we suggest a model for predicting FPG after supplementation with vitamin D. We showed that the effect of vitamin D was to reduce FPG 30% (Tables 4,5,6).

**Table 4:** Model for prediction of final FPG after treatment with vitamin D for 8 weeks in 100 patients with type 2diabetes

**Model**	**Unstandardized coefficients**	**Standardized coefficients**	**Sig**	**R Square change**
	**B**	**Beta**		
Constant	0.337		0.991	
FBS-1	0.460	0.434	0.00	0.032
Vitamin D	−0.394	−0.195	0.022	
gender	16.488	0.195	0.020	
HbA1C	7.024	0.184	0.031	

**Model**: **FBS**_**2**_ = 0.46 × FBS_1_ 0.39×Vitamin D+16.4×Gender +7.02×HbA_1_C+ 0.33.

**FPG1:** Baseline fasting plasma glucose.

**Gender**:male 1, Female 2.

**Vitamin D:** Baseline 25(OH)D.

**Table 5:** Model for prediction of fasting serum Insulin after treatment with vitamin D in 100 patients with type 2 diabetes for 8 weeks

**Model**	**Unstandardized coefficients**	**Standardized coefficients**	**Sig**	**R Square change**
	**B**	**Beta**		
Constant	9.332		0.000	
Insulin 1	0.258	0.296	0.002	0.036
25(OH) D	−0.082	−0.191	0.048	

**Model**: **Insulin 2** = 0.25 × Insulin 1 0.08 × Vitamin D + 9.33.

**Vitamin D:** Baseline 25(OH)D.

**Insulin1 :** Baseline insulin.

**Table 6:** Model for prediction of HOMA-IR after treatment with vitamin D

**Model**	**Unstandardized coefficients**	**Standardized coefficients**	**Sig**	**R Square change**
	**B**	**Beta**		
Constant	3.432		0.00	
HOMA_IR_1	0.276	0.268	0.006	0.043
Vitamin D	−0.035	−0.208	0.031	

**Model**: **HOMA-IR 2** = 0.27 × HOMA-IR 1 0.03 × Vitamin D + 3.43.

**Vitamin D:** Baseline 25(OH)D.

**HOMA-IR-1:** Baseline HOMA-IR.

## Discussion

The main purpose of this study was to investigate the effects of vitamin D supplementation on glucose homeostasis. The results showed that vitamin D supplementation significantly decreased serum FPG,insulin and HOMA-IR in patients with T2DM.

The role of basal serum FPG and vitamin D, sex, and HbA1c in predicting FPG final was 36%. There was an interesting finding. There was an inverse relation between final FPG and basal 25(OH) D concentration. In other words, higher serum basal 25(OH) D led to lower final FPG. This means that who had a higher serum basal 25(OH) D concentration benefited more of vitamin D intake to lowering final FPG. This may be because of non-skeletal effects of vitamin D which appears in higher vitamin D concentration and the effects of lower vitamin D concentration, are limited to the bone and muscle. Our data showed that effects of vitamin D on insulin resistance was significant when vitamin D concentration was 40–60 ng/ml(100–150 nmol/l) and in lower and upper vitamin D concentration, it didn,t affect on insulin resistance.

Effects of vitamin D supplementation on glucose homeostasis have been shown in numerous studies. Our findings are consistent with results of many other published studies, in which the insulin resistance appears to be decreased in T2DM patients who had received vitamin D. For example,Inzucchi showed a 60% improvement in insulin sensitivity by increased serum 25 (OH)D concentration from 10 to 30 ng/ml(25 to75nmol/l), by which metformin or troglitazone were 54% and 13% respectively [14]. Von Hurst (2009) showed that vitamin D supplementation significantly improved insulin sensitivity and insulin resistance [21]. Ken (2004) found an inverse relation between 25(OH) vitamin D concentration and FPG, but a direct relation with insulin sensitivity [17].

As to these studies, our study shows that mean FPG was significantly reduced after increased vitamin D intake. Monthly supplementation with 120,000 units of vitamin D also improved insulin sensitivity [22]. Although in contrast to some studies, there was a significant reduction in HOMA-IR after taking vitamin D, Witham foundout that vitamin D intake (at different dosage) had no effects on insulin resistance or on HbA1c [23] as did Lind [24]. Nagpal reported that vitamin D supplementation had no effect on mean of insulin sensitivity but two years treatment with vitamin D did improve HOMA-IR [25].

There are some mechanisms for the effects of vitamin D: presence of vitamin D receptors on pancreatic β cells [1], Vitamin D activating 1α hydroxylase is expressed in pancreatic β cells [26], presence of vitamin D response element in the insulin gene [27], presence of vitamin D receptor in skeletal muscle [28] and the fact that 1,25(OH)D increases transcription of insulin receptor genes [13], and also suppresses the renin gene reducing hyperglycemic-induced increases in renin levels in pancreatic β cells and blockade of renin-angiotensin activity has been proposed as a novel target for diabetes treatment [29].

Protective effects of vitamin D on diabetes,maybe due to well known effects of vitamin D such as its anti-inflammatory properties, its effects on calcium and phosphorus metabolism and regulation of the insulin receptor gene [13]. It seems that vitamin D increases in calcium content of the cells, in turns leading to increased transport of glucose into the muscle [30]. Vitamin D also regulates nuclear PPAR (Peroxisome proliferative activated receptor)that has an important role in the insulin sensitivity [31]. vitamin D deficiency is associated with increases in inflammation. Vitamin D attenuates the expression of proinflammatory cytokines involved in insulin resistance such as interleukins, IL-1, IL-6, TNF-a, also down regulates NF-Kb (Nuclear factor) activity [32].

It would be useful, though to undertake further studies to discover more about the mechanism and the effect of vitamin D on both alpha and islet beta-cell function and also on the mechanisms determining insulin resistance.

Some Iranian studies also showed that calcitriol [33],vitamin D injection [34] and vitamin D intake could,nt affect on diabetes and insulin resistance [35]. Although some of them reported significant effects of vitamin D on diabetes [36].

A limitation of our study is that we did not evaluate the effects of placebo on FPG, insulin or HOMA-IR. However, there are a few studies that didn’t use placebo, evaluated the effects of vitamin D at different doses on glucose homeostasis [21].

## Conclusion

It seems that vitamin D can improve diabetes control and it is recommended that vitamin D supplementation should be included in treatment of type 2 diabetes.

## Competing interests

The authors declare that they have no competing interests.

## Authors’ contributions

AT carried out the design of the study, participated in the coordination of the study and drafted the munscript.MM performed the statistical analysis and helped to draft the manuscript.ZA participated in the design of the study and helped to draft the manuscript. All authors read and approved the final manuscript.
